# CSF Biomarkers of Neurodegeneration in Progressive Non-fluent Aphasia and Other Forms of Frontotemporal Dementia: Clues for Pathomechanisms?

**DOI:** 10.3389/fneur.2018.00504

**Published:** 2018-07-02

**Authors:** Peter Körtvelyessy, Hans J. Heinze, Johannes Prudlo, Daniel Bittner

**Affiliations:** ^1^Department of Neurology, University Hospital Magdeburg, Magdeburg, Germany; ^2^German Center for Neurodegenerative Diseases, Magdeburg, Germany; ^3^Leibniz Institute for Neurobiology, Magdeburg, Germany; ^4^German Center for Neurodegenerative Diseases, Rostock, Germany; ^5^Department of Neurology, University Hospital Rostock, Rostock, Germany

**Keywords:** frontotemporal dementia, cerebrospinal fluid, phospho-tau, tau, amyloid beta, progranulin, neurofilament light chain

## Abstract

Frontotemporal Dementia (FTD) encompasses distinct pathophysiologically heterogenous disorders with different genetic and cellular disease mechanisms. The objective of this study is to compare the constellation of biomarkers of neurodegeneration in the cerebrospinal fluid (CSF) to the FTD type categorized by clinical symptoms. We investigated the levels of Phospho_181_-tau, Total-tau, Beta-amyloid_1−42_, Neurofilament light chain, and Progranulin in the CSF of *n* = 99 FTD patients regarding to the different subtypes of FTD, including semantic dementia (SD), progressive non-fluent aphasia (PNFA), behavioral variant FTD (bvFTD). We compared these groups to patients without neurodegenerative disorders and another cohort encompassing tauopathies with distinct clinical syndromes (Cortico basal syndrome and progressive supranuclear palsy) and logopenic PNFA (lPPA) as another disorder with predominant speech disturbance. CSF-Progranulin levels were significantly lower in FTD type patients with semantic dementia and behavioral variant FTD mainly attributed to the Tar-DNA-Binding-Protein (TDP) 43 compared to predominantly Tau-mediated PNFA (*p* < 0.05). Also, neurofilament light chain was significantly higher (*p* < 0.036) in all FTD patients especially in SD patients (*p* < 0.01). CSF-Nfl levels also distinguished SD patients from logopenic Alzheimers patients (*p* < 0.05). In sum, CSF-Neurofilament light chain and CSF-Progranulin seem to be promising biomarkers for FTD, the latter predominantly for assumed TDP43-mediated FTD.

## Introduction

Frontotemporal dementia is the second most common form of dementia in patients under the age of 65 (1). In a subgroup of patients with semantic dementia (SD) histopathologically, Tar-DNA-Binding-Protein 43 (TDP-43) aggregates are the main characteristic of a subgroup of patients with semantic dementia (SD) (2), and pathomechanisms associated with dysfunctional TDP-43 or TDP-43 aggregates could play a major role in this form of the disease. Similar observations have been made in most behavioral type (bv)FTD and especially in FTD associated with a concomitant motoneuron disease (3). In contrast, progressive non-fluent aphasia (PNFA) appears predominantly associated with tau-pathology (4, 5). Recent reports have provided evidence that alterations in the *GRN* gene are associated with PNFA in rare cases (6). There is also a logopenic variant (lPPA) of PNFA which is closely related to the Alzheimer's dementia (AD) although not exclusively (7), potentially mimicking an FTD. CSF measurements of biomarker of neurodegeneration are now part of the clinical routine with an excellent sensitivity for Alzheimer pathomechanisms (8). The diagnosis of a FTD in clinical routine is more challenging than the diagnosis of an Alzheimer's dementia (AD). Till today no biomarker or biomarker constellation for FTD has been established. In sum, there is a need for a biomarker for the different FTD and especially for the different forms of FTD. CSF-Progranulin (PGRN) and Neurofilament light chain (Nfl) are promising candidates as biomarkers for TDP-43 mediated neurodegeneration in Amyotrophic lateral sclerosis (3, 9) and are therefore interesting candidates in TDP-43 mediated FTD.

Cerebral PGRN is a major pathophysiologic component in TDP43-aggregates associated forms of neurodegeneration (10). However, there is little knowledge on whether reduced CSF-PGRN levels are of pathophysiological and/or diagnostic relevance in TDP43-mediated FTD without *GRN* deficiency. Reduced PGRN levels are observed in patients suffering from a *GRN* mutation, exhibiting both low serum and CSF levels of PGRN (11). It has been shown that different single nucleotide polymorphisms are important for CNS and serum PGRN metabolism, respectively (12). In consequence, the cerebral metabolism of PGRN needs to be assessed via CSF and not via serum analyses (12, 13). In line with this observation, a previous study by our group showed a significant difference between CSF-PGRN levels in AD and FTD patients, while serum levels did not differ (14). This was confirmed by Wilke et al. (15), who found lower CSF-PGRN levels in FTD patients without *GRN* mutations. Lower CSF-PGRN levels also correlate with disease duration in TDP43-associated Amyotrophic Lateral Sclerosis (ALS) (16). Therefore, we have determined PGRN levels in the CSF of patients with different forms of FTD and compared them with other forms of dementia in this study to investigate whether different CSF biomarker constellations mirror the supposed histopathological differences in FTD patients and especially differences between TDP43-mediated FTDs on one hand and the Tau-/ Fused in Sarcoma Protein (FUS)-mediated FTDs on the other.

We also examined CSF-Nfl levels in all cohorts to determine whether CSF-Nfl levels may be a biomarker for TDP-43 mediated FTD patients. Neurofilaments are proteins inside the cytoskeleton in central and peripheral neurons (8). The appearance of Nfl in the CSF is strongly associated with axonal dysfunction and subsequent neurodegeneration, especially in ALS (9).

## Materials and methods

The local ethics committees at the University Hospitals Magdeburg and Rostock approved this study and every patient gave written and informed consent.

### Cohorts

FTD-patients were clinically diagnosed according to the Neary et al. consensus criteria (17) at the tertiary dementia centers of the German Center for Neurodegenerative Diseases (DZNE) in Magdeburg, Germany and in Rostock, Germany. The lumbar puncture was performed as part of the first clinical work up of these dementia patients. Therefore the patients are not in an advanced phase of their disease. We included 99 patients (mean age 67.6 ± 8.2 years) with FTD. Forty-four patients have been diagnosed with bvFTD, 33 with SD (together *n* = 77 non-PNFA) and 22 with PNFA. We have included several control groups from Magdeburg, Germany. One group with patients suffering from other neurological diseases without neurodegeneration e.g., acute headache or patients in which infectious diseases were excluded (*n* = 39; mean age = 66.3 ± 9.8 years). The other control group comprises patients suffering from cortico basal syndrome (CBS) and progressive supranuclear palsy syndrome (PSP) (*n* = 18, mean age = 70.8 years). Both syndromes have a tau-mediated pathomechanism (2). Both control groups are age-matched to the FTD cohort. Finally a control group with a predominant speech disorder, lPPA (*n* = 15) was included. Diagnosis was obtained via neuropsychological testing and patient's history. Mean age was higher than in all other groups (72.7 ± 7.5 years, *p* < 0.05). We excluded patients with known *GRN*-mutations. The proportion of men and women was nearly equal in all cohorts except in the SD cohort (30.3% men) with a female preponderance (see Table 1).

**Table 1:** Demographic data and biomarker levels for the diagnostic groups.

**Characteristic^a^**	**FTD (*n* = 99)**	**PNFA (*n* = 22)**	**bvFTD (*n* = 44)**	**SD (*n* = 33)**	**CBD/PSP (*n* = 18)**	**Controls (*n* = 39)**
No. (%) of men	51 (51.5)	14 (60.9)	28 (63.6)	10 (30.3)	10 (55.6)	23 (58.6)
Age (years)	67.6 ± 8.2	66.8 ± 7.5	67.9 ± 9.2	67.7 ± 7.4	70.8 ± 4.6^b^	66.3 ± 9.8
p-Tau, pg/ml (>70 ng(ml)	60.8 ± 28.8^c^^,^ ^e^	62.6 ± 23.8	52.0 ± 23.2	70.2 ± 34.4^f^^,^ ^h^	42.9 ± 16.2	51.5 ± 19.1
t-Tau, pg/ml (>350 ng/ml)	407 ± 233^b^^,^ ^d^	413 ± 223^e^	344 ± 215	479 ± 244^e^^,^ ^h^	250 ± 150	283 ± 139
Aβ1-42, pg/ml (< 485 ng/ml)	712 ± 304^b^	824 ± 347	712 ± 284^b^	652 ± 295^b^	631 ± 200^b^	1007 ± 305
PGRN, pg/ml (0.72 < >1.16 ng/ml)	0.84 ± 0.18^e^	0.99 ± 0.19	0.80 ± 0.15^f^^,^ ^g^	0.78 ± 0.14^f^^,^ ^g^	0.96 ± 0.23	0.94 ± 0.22
Nfl, pg/ml (>3643 ng/ml)	4808 ± 3082^b^	4851 ± 2763^e^	3821 ± 2753	6477 ± 3370^b^^,^ ^h^	na	2035 ± 1395

*Pathological levels are in brackets. FTD, frontotemporal dementia; CBD, corticobasal degeneration; PSP, progressive supranuclear palsy; bvFTD, behavioral variant frontotemporal dementia; SD, semantic dementia; PNFA, primary non-fluent aphasia; Aβ1-42, β-amyloid 1-42; Nfl, neurofilament light chain; t-Tau, total-Tau; p-Tau, phospho_181_-Tau; PGRN, Progranulin*.

a *data given as mean ± standard deviation unless otherwise indicated. Statistical differences were determined using multivariate analysis with post-hoc tests. Only statistically significant results are noted*.

b *Compared with controls, P < 0.001*.

c *Compared with CBD/PSP, P < 0.05*.

d *Compared with CBD/PSP, P < 0.01*.

e Compared with controls, P < 0.05

f *Compared with controls, P < 0.01*.

g *Compared with PNFA, P < 0.001*.

h *Compared with bvFTD, P < 0.05*.

### CSF-measurement

All biomarkers were measured in Magdeburg, Germany. The patients from Rostock were measured in a batch. The biomarkers from the patients from Magdeburg were prospectively measured as part of the clinical routine. With each assay, the clinical samples were run together with a blank (sample diluent), the (prepared) calibrator solutions and the appropriate control. All samples were run in duplicate. The arithmetic mean was always taken as final result. The operators were blinded for patient's characteristics. Phospho-tau (p-tau), Total-tau (t-tau), and Aβ_1−42_ were measured in CSF from 18 PNFA patients, from 72 non-PNFA patients and the 15 lPPA. CSF-PGRN was measured in 22 PNFA-patients, 14 lPPA and 77 non-PNFA patients. CSF-Neurofilament light chain (Nfl) was measured in 15 lPPA, 15 PNFA patients and 41 non-PNFA patients.

CSF was obtained during routine clinical diagnostics, processed within 30 min after puncture and then stored at −80°C. CSF was processed according to the manufacture's instructions for each ELISA Kit. CSF levels of Aβ_1−42_, total-tau, and tau_181P_ were measured with commercially available single-parameter ELISA kits [respectively Innotest β-amyloid (1–42), Innotest hTauAg, Innotest phospho-Tau(181P), Innogenetics, Ghent, Belgium]. For PGRN-measurement CSF-samples were diluted 1:3, and an ELISA was performed to determine the levels of PGRN in the CSF (Human Progranulin ELISA Kit, Mediagnost, Reutlingen, Germany). The sensitivity of this assay was 18 pg/ml. A cut-off was determined at 0.72 ng/ml and at 1.16 ng/ml, which equals 1 standard deviation below and above the mean of our control sample with other neurological disease (0.94 ± 0.22 ng/ml). This narrow margin was chosen because patients suffering from a GRN gene mutation had CSF-PGRN levels as low as 2 standard deviations below normal levels (6).

CSF-Nfl was measured with a commercial ELISA (by Umandiagnostics, Sweden). The sensitivity of this assay was 31 pg/ml. The cut-off was at 3643 pg/ml (mean healthy control group + two standard deviations).

Statistical analysis was performed using SPSS 21.0 package. For categoric variables Chi-square test was applied. To assess in a first step differences between FTD patients as a whole group and controls, a two-sample *t*-test was used. Comparing interval-scaled variables between all groups a MANOVA test was performed with age and gender as confounding parameters followed by subsequent Bonferroni *post-hoc* analysis. All statistical tests were two-sided. Significance level was set at 0.05.

## Results

In order to test whether different forms of FTD are associated with differences in CSF biomarker constellation and in particular with differences in levels of PGRN, we compared these biomarkers in 171 patients, among them 39 control individuals without neurodegenerative disorders, 44 patients with bvFTD, 33 patients with SD, 22 patients with PFNA, and 15 lPPA patients. An additional group of 18 patients with CBS/PSP suffered from neurodegeneration based on tau pathology and served as a disease control group.

There were no group differences overall concerning basic demographics but age, that was higher in the CBS/PSP group compared to controls (*p* < 0.001) and gender in SD with a female preponderance (*p* = 0.012).

In a first step differences of CSF markers between FTD and controls were analyzed using a two sample *t*-test. In FTD patients Tau_181p_ (*p* < 0.05), t-tau (*p* < 0.001), and Nfl (*p* < 0.001) were higher, Aβ_1−42_ (*p* < 0.001) and PGRN lower (*p* = 0.013; Table 1).

**Figure 1:**
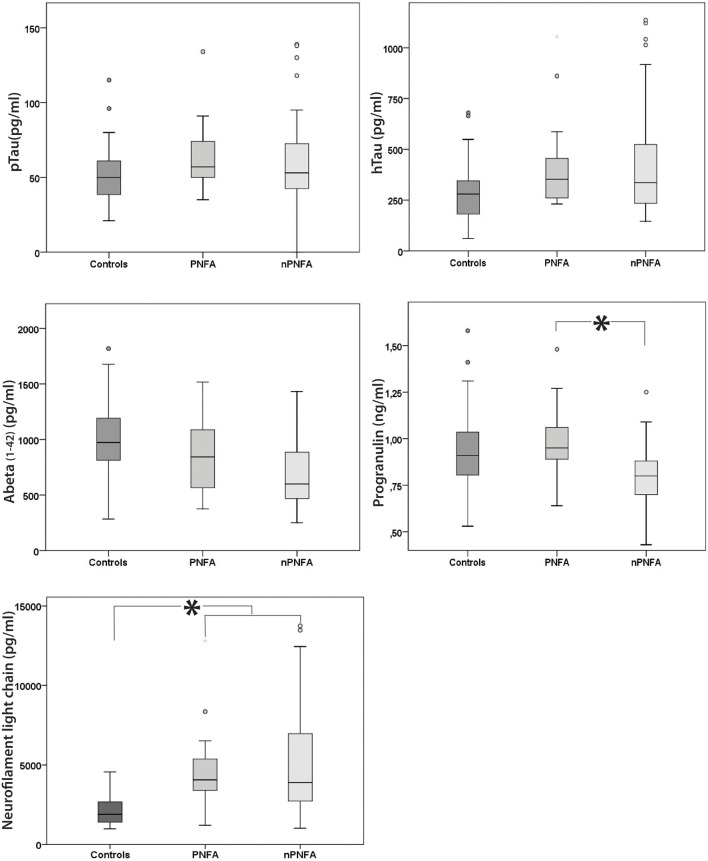
Box plot of the different biomarkers in the PNFA and nonPNFA Group (SD and bvFTD) compared to our controls; PNFA = progressive non-fluent Aphasia; nPNFA = non PNFA; ^*^meaning *p* ≤ 0.001 // one-way ANOVA showed: Phospho181-Tau PNFA vs nPNFA *p* = 0.952 (*F* = 0.058); Total-tau PNFA vs nPNFA *p* = 0.748 (*F* = 0.204); Amyloid beta (1–42) PNFA vs nPNFA *p* = 0.214 (*F* = 1.241); PGRN PNFA vs nPNFA *p* ≥ 0.001^*^ (*F* = 0.183); Nfl PNFA vs nPNFA *p* = 0.91 (*F* = 0.817). Circles are marking outliers and the star extreme outlier.

In a second step the groups were compared in more detail. PGRN in patients with SD and bvFTD was significantly lower compared to PNFA (*p* < 0.001) and controls (*p* < 0.01; Figure 1, Table 1). The comparison of PGRN in both predominantly tau-mediated cohorts, the CBD/PSP group and PNFA patients, revealed no significant differences (*p* > 0.05).

Differences of Total-tau were mainly attributed to higher levels in SD patients ([T-tau] = 479 pg/ml), compared to controls (*p* < 0.05) and CBD/PSP (*p* < 0.05) (see Table 1). Tau_p181_ was higher comparing all FTD patients to CBD/PSP patients (*p* < 0.05) (see Table 1) but still within the normal range ([Tau_p181_] = 60.8 pg/ml with a cutoff at >70 pg/ml). It was further higher in SD compared to controls (*p* < 0.01) and bvFTD (*p* < 0.05).

Overall, Aβ_1−42_ levels were within the physiological range (cutoff > 485 pg/ml) in all patients and never reached pathologically low levels as in AD except the lPPA group. Aβ_1−42_ on the other hand was significantly lower in SD, bvFTD, and CBS/PSP patients than in our controls (*p* < 0.001; Table 1).

Nfl was higher in the SD group compared to controls (*p* < 0.001) and bvFTD (*p* < 0.05), and in PNFA compared to controls (*p* = 0.05).

Comparison of biomarkers of neurodegeneration in PNFA vs. non-PNFA patients showed no differences for Tau_181p_ (*p* = 0.7), for t-tau (*p* = 0.9), for Aβ_1−42_ (*p* = 0.082), and for Nfl (*p* = 0.9) (see Figure 1).

Finally the FTD groups with predominantly speech disturbance (SD and PNFA) were analyzed including the group with lPPA. The latter had an Alzheimer-like CSF status (see Table 2) resulting in a significantly higher Tau_181p_ (*p* = 0.05) and significantly lower Aβ_1−42_ level (*p* < 0.01) compared to PNFA. Abeta ratio (1-42/1-40) was lower in the lPPA patients when compared to SD (*p* < 0.05). PGRN was not different in the lPPA group compared to the PNFA group (see Table 2), while CSF-Nfl was significantly lower in the lPPA group (*p* < 0.05) compared to the SD group.

**Table 2:** Comparison of logopenic PPA directly with non-fluent progressive Aphasia and semantic dementia.

	**logopenic PPA (*n* = 15)**	**non-fluent progressive aphasia (*n* = 22)**	**Semantic dementia (*n* = 33)**
Age (years)	72.7 ± 7.5	66.8 ± 7.5	67.7 ± 7.4
pTau (pg/ml)	91 ± 37^a^	63 ± 24^a^	70 ± 34
hTau (pg/ml)	594 ± 282	413 ± 223	479 ± 244
Aβ_1−42_ (pg/ml)	480 ± 182^b^	824 ± 347^b^	651 ± 295
Abeta ratio	0.61 ± 0.29^c^	0.88 ± 0.37	1.02 ± 0.57^c^
PGRN (ng/ml)	0.90 ± 0.21	0.99 ± 0.19^d^	0.78 ± 0.14^d^
Nfl (pg/ml)	3751 ± 1090^c^	4852 ± 2763	5027 ± 2776^c^

a *p = 0.05 logopenic PPA vs. non-fluent Aphasia*.

b *p < 0.01 logopenic PPA vs. non-fluent Aphasia*.

c *p < 0.05 logopenic PPA vs. semantic dementia*.

d *p < 0.001 non-fluent aphasia vs. semantic dementia*.

## Discussion

FTD comprises a heterogenous group of disorders, which are predominantly diagnosed according to clinical parameters. Despite clear distinctions based on postmortem neuropathological parameters, no biomarkers have been defined yet which could help to distinguish these different disorders (2). The heterogeneous pathologies summarized under the term FTD might account for the so far not promising results when looking for a typical biomarker constellation in all FTD patients without *GRN* mutations as was done previously (13–15, 18, 19). Our data indicate that the PGRN-levels differ in the CSF between subgroups of FTD, supposedly mirroring the different pathological mechanisms and allowing to separate PNFA from SD/bvFTD (4, 20, 21). Lower CSF-PGRN might mirror the change in cerebral TDP-43 metabolism in patients with TDP 43 pathology (10) as already seen in patients suffering from mutations in the *GRN* gene, but to a smaller extent. Similar observations have recently been reported by others who found a significantly lower CSF-PGRN level in GRN-mutation negative FTD patients, but they did not differentiate FTD by the different etiologies nor by dominant clinical symptoms therefore not taking into account the heterogeneous picture of FTD resulting in potentially different biomarker constellation(15). The predominantly tau-mediated PNFA and our disease control group comprising solely tau-mediated diseases showed significantly higher (normal) CSF-PGRN levels probably because TDP-43 mechanisms were not centrally involved in pathophysiology in these diseases. In contrast, e.g., patients suffering from a semantic dementia, which represents a mainly TDP-43 mediated FTD had low CSF-PGRN levels again reflecting the central pathomechanism (see Table 1) (20, 22). Still, the lower CSF-PGRN levels were not outside the normal range but at the bottom end, attenuating this result. lPPA did show a mostly Alzheimer-like CSF constellation and no signs of an alteration in the cerebral PGRN/TDP-43 metabolism (see Table 2) (23).

It is well-known that tauopathies such as CBS or PSP cannot be diagnosed via CSF-tau_p181_ (24). Even in FTD patients with a tau mutation, no alteration of CSF-tau was found (25). It is therefore not surprising that our cohort of PSP/CBS patients did neither reveal a significant difference when compared to the mainly tau-mediated PNFA group or to the control groups. The pathologically elevated t-tau levels in FTD patients have been described before (26, 27) but no distinction has been made between SD and bvFTD patients as we could do here.

Considering other biomarkers, we could confirm previous results by other groups showing a highly significant difference between Neurofilament light chain levels in general in FTD patients in comparison to controls with other neurological diseases (27). When adjusting for the different clinical subtypes only SD and PNFA have significantly higher CSF-Nfl levels and not bvFTD (28, 29).

There are discrete differences in clinical symptoms of an FTD such as the presence and extent of disease defining symptoms in bvFTD. This could contribute to a potential heterogeneity of our bvFTD group. In contrast, the diagnosis of a SD with its dominant agrammatical aphasia is easier to make. This heterogeneity is probably responsible for the slightly higher PGRN levels in the bvFTD group compared to the more homogenous SD group.

Another potential limitation of this study is the lack of differentiation in terms of apraxia of speech in our PNFA group since this clinical symptom might be due to an underlying tauopathy (30).

A more precise categorization of patients in the PNFA and the other FTD groups was unfortunately not possible in this retrospective study. Applying additional filters could help to extend the results found in our study. On the other side, a distinction between supposed FUS-mediated FTD types and TDP-43 mediated FTDs could not be performed due to the lack of clinical differentiation in our FTD cohort.

These results are a further step toward validating CSF-Neurofilament light chain as biomarker in FTD in general and especially in the PNFA and SD clinical phenotypes. CSF-PGRN might be a marker for FTD-TDP43 and probably other TDP-43 mediated diseases. CSF-PGRN needs further validation via combined histopathological and clinical studies.

We recommend to measure Nfl and PGRN in the diagnostical work up of dementias.

## Author contributions

PK and DB study design, acquisition of data, data analysis, medical writing. JP revising the manuscript for content, acquisition of data. HH revising the manuscript for content.

### Conflict of interest statement

The authors declare that the research was conducted in the absence of any commercial or financial relationships that could be construed as a potential conflict of interest.

## Abbreviations

Aβ_1−42_: Amyloidbeta 1-42
bvFTD: behavioral variant Frontotemporal Dementia
CBS: corticobasal syndrome
CSF: Cerebrospinal fluid
FUS: fused in sarcoma protein
FTD: Frontotemporal Dementia
*GRN*: Granulin
lPPA: logopenic Variant of primary progressive aphasia
Nfl: Neurofilament light chain
PGRN: Progranulin
PNFA: progressive non-fluent aphasia
PSP: Progressive supranuclear palsy
SD: semantic dementia
TDP43: tar-DNA-binding-protein 43
Tau_p181_: Tau_phospho181_
t-tau: Total tau.
